# Endoplasmic reticulum stress activation in adipose tissue induces metabolic syndrome in individuals with familial partial lipodystrophy of the Dunnigan type

**DOI:** 10.1186/s13098-017-0301-6

**Published:** 2018-02-09

**Authors:** Maria C. Foss-Freitas, Rafael C. Ferraz, Luciana Z. Monteiro, Patricia M. Gomes, Ricardo Iwakura, Luiz Carlos C. de Freitas, Milton C. Foss

**Affiliations:** 1 Department of Medicine, Division of Endocrinology and Metabolism, School of Medicine of Ribeirao Preto, University of Sao Paulo, Ribeirao Preto, São Paulo Brazil; 2 University of Brasilia-UNB, Federal District, Brasilia, Brazil; 3 Department of Opthalmology, Otolaryngology, Head and Neck Surgery, School of Medicine of Ribeirao Preto, University of Sao Paulo, Ribeirao Preto, São Paulo Brazil

**Keywords:** Familial partial lipodystrophy, Dunnigan type, Endoplasmic reticulum stress, Insulin resistance

## Abstract

**Background:**

Familial partial lipodystrophy of the Dunnigan type is one of the most common inherited lipodystrophies variables. These individuals have important metabolic disorders that cause predisposition to various diseases. In this study we aimed to demonstrate the relation between the metabolic abnormalities, inflammatory profile and the expression of genes involved in the activation of the endoplasmic reticulum stress (ERS) in subjects with FPLD.

**Methods:**

We evaluated 14 female FPLD patients and compared with 13 female healthy individuals. The subjects were paired with their respective BMI and age and categorized into two groups: Familial partial lipodystrophy of the Dunnigan type (FPLD) and control. Patients were fasted for 12 h before blood collection for measurement of HbA1c, glucose, insulin, lipids and inflammatory markers. Subcutâneous adipose tissue was collected by puncture aspiration of submental region during ambulatorial surgical aesthetic procedure.

**Results:**

We demonstrate that patients with FPLD show increased HbA1c (p < 0.01), fasting glucose (p < 0.002) and triglycerides (p < 0.005) while HDL/cholesterol (p < 0.001) was lower when compared to healthy individuals. We found that 64.2% FPLD patients had metabolic syndrome according to International Diabetes Federation definition. We also observe increased AUC of glucose (p < 0.001) and insulin during oGTT, featuring a frame of hyperglycemia and hyperinsulinemia, suggesting insulin resistance. Also we found hyperactivation of several genes responsible for ERS such as ATF-4 (p < 0.01), ATF-6 (p < 0.01), EIF2α3K (p < 0.005), CCT4 (p < 0.001), CHOP (p < 0.01), CALR (p < 0.001) and CANX (p < 0.005), that corroborate the idea that diabetes *mellitus* and metabolic syndrome are associated with direct damage to the endoplasmic reticulum homeostasis. Ultimately, we note that individuals with lipodystrophy have an increase in serum interleukins, keys of the inflammatory process, as IL-1β, TNF-α and IL-6 (p < 0.05 all), compared with healthy individuals, which can be the trigger to insulin resistance in this population.

**Conclusion:**

Individuals with FPLD besides having typical dysfunctions of metabolic syndrome, show a hyperactivation of ERS associated with increased systemic inflammatory profile, which together may explain the complex clinical aspect of this diseases.

*Trial registration* HCRP no 6711/2012

**Electronic supplementary material:**

The online version of this article (10.1186/s13098-017-0301-6) contains supplementary material, which is available to authorized users.

## Background

Lipodystrophy are part of a clinically heterogeneous group of inherited or acquired diseases characterized by selective or total loss of adipose tissue. Affected patients are predisposed to insulin resistance and the development of related diseases such as visceral obesity, hypertension, type 2 diabetes mellitus, metabolic syndrome, dyslipidemia, coronary artery disease and hepatic steatosis [1, 2]. Recently, the diagnosis of lipodystrophy has been associated with increased morbidity and mortality in patients seropositive for human immunodeficiency virus (HIV) on antiretroviral treatment [3, 4]. The molecular mechanisms involved in insulin resistance and the metabolic complications presented by patients with lipodystrophy, especially in hereditary forms are not clearly understood [5]. Being essentially clinically diagnosed, these diseases are considered rare and little known, and may go unnoticed in daily clinical practice.

The Familial partial lipodystrophy of the Dunnigan type (FPLD) is the most common and well characterized genotype–phenotype between hereditary forms of lipodystrophy, it is associated with insulin resistence and can evolve with decreased glucose tolerance and development of diabetes mellitus and dyslipidemia [6]. It is an autosomal dominant disorder, resultant of mutations in the LMNA gene, in which the loss of subcutaneous fat affects the extremities, buttocks area, abdomen and torso. Fat deposits tend to accumulate on the face, neck and submental regions, supraclavicular and intra-abdominal. In women, the frequent presence of hirsutism, menstrual abnormalities, absence of obesity of the trunk and the accumulation of fat in the genital area facilitates the diagnosis [6, 7]. Recent studies have shown that the expression of the phenotype varies according to gender, with men presenting less aggressive metabolic profile than female patients [6].

There is growing evidence that endoplasmic reticulum stress (ERS) activation is a central feature involved in the pathogenesis of a variety of diseases, including diabetes mellitus [8]. The activated ERS is responsible for inducing apoptosis to be able to contribute to insulin resistance [9]. In addition, ERS is the consequence of a lack of control in the amount of poorly folded proteins inside the ER, which, in turn, to restore homeostasis, the cell develops a response system known as depleted protein response (UPR) [10]. The cellular imbalance induced by UPR activation interferes with the calcium balance in ER affecting genes such as calreticulin (CARL) and calnexin (CANX) [11, 12]. Several mechanisms are the mechanisms by which UPR reverses ERS, including the transmembrane sensor, such as the eukaryotic 2-alpha kinase 3 translation initiation factor (EIF2AK3 or PERK), when activated by UPR, are sufficient to activate the factor of transcription activator 6 (ATF-6), resulting in a reduction of protein synthesis avoiding the accumulation of new unfolded proteins [13]. ERS plays an important role in cellular functioning, however, some studies have shown that ERS is involved in beta cell dysfunction of the pancreas, which are responsible for insulin production [14]. There are few studies evaluating ERS in patients with lipodystrophy, but recently it has been reported that accumulation of prelamin A causes mitochondrial dysfunction, endoplasmic reticulum stress and altered lipid metabolism, similar to a phenotype of premature aging [15]. On the other hand, once the involvement of ERS in lipodystrophy has been established, new drugs and interventions that are capable of reversing the insulin resistance or dyslipidemia, frequently observed in these diseases, can be investigated [16].

Despite the reduction in the amount of adipose tissue disposed through the body of these individuals, the increased prevalence of the metabolic syndrome is evident. Many studies have shown that pro-inflammatory cytokines, produced by adipocytes, reduce insulin sensitivity induced by interference in the insulin signaling pathway [17]. In addition to the inflammatory process, the activation of endoplasmic reticulum stress, particularly in adipose tissue, plays an important role in the development of insulin resistance and hyperglycemia [8].

In this study, we analyzed metabolic and inflammatory profile and also the expression of some important genes involved in the activation of ERS, which plays an important role in insulin resistance and dislipidemia, in subjects with FPLD.

## Methods

### Individuals

After obtaining the consent, 14 female patients with FPLD, followed at the Diabetes Outpatient Clinic, and 13 female healthy subjects paired for age and BMI, constituting the control group, were studied. Subjects with FPLD had clinical and molecular testing diagnosis as described previously [18]. The study was approved by the Research Ethics Committee (HC-FMRP-USP—Process HCRP no 6711/2012) of the Institution and all volunteer subjects gave written informed consent to participate.

### Inclusion criteria

All patients in the FPLD group had androgenic phenotype, muscular hypertrophy, increased waist/hip ratio (> 0.85), breast hypotrophy, hyperinsulinemia, rounded and wide facies, fat deposition in the cervical and mandibular regions, filled supraclavicular fossa, acanthosis nigricans, menstrual irregularity, DM and hyperlipidemia. The FPLD diagnosis was confirmed by molecular testing that was previously described [18]. Control group volunteers were selected aleatory in the outpatient clinic, they should not present the above characteristics but should be matched for age and BMI with FPLD subjects.

### Exclusion criteria

Patients who had chronic DM complications in advanced stages (proliferative retinopathy, chronic renal failure, autonomic neuropathy, and acute myocardial infarction or history of peripheral vascular accident less than 6 months), gestation, chronic or acute liver diseases, cancer and high levels of plasma LDL cholesterol (above 150 mg/dL) were not enrolled in the study.

### Use of medications

All subjects in the FPLD group were using metformin and statins to control glycemic and cholesterol levels. Healthy subjects in the control group were not using any medication.

### Data collect

Patients and healthy volunteers were submitted initially to clinical evaluation and body composition, weight, height and waist circumference, as well as oGTT. There were also collected blood samples for glucose, insulin and lipid profile measurements, for this procedure patients and volunteers were fasted for 8 h.

### Specific laboratory determinations

A blood sample was collected after an overnight fast of 8 h. Plasma glucose levels were measured using COBAS INTEGRA 400 plus (Roche, USA). Fasting insulin levels were measured using a radioimmunoassay by Immulite I (Siemens, USA). Fasting lipids were analyzed, and for the present study serum levels of total cholesterol, LDL, HDL and triglyceride using COBAS INTEGRA 400 plus (Roche, USA).

### Insulin resistance evaluation and systemic inflammatory state

Serum levels of CRP were evaluated for systemic inflammatory evaluation, as well as HbA1c and lipids and total fractions. At the same time, serum insulin and fasting blood glucose levels were measured to calculate the HOMA index. The method is based on basal glycemia and fasting insulin to assess insulin resistance (HOMA-IR) and β-cell function (%β). The following equations were used for this calculation:

$${\text{HOMA-}}\upbeta = \frac{{{20} \times {\text{Basal}}\,{\text{insulin}}\,(\upmu {\text{U/mL)}}}}{{{\text{Fasting glycemia}}\, ( {\text{mmol/L)}}-3.5}}$$

$${\text{HOMA-IR}} = \frac{{{\text{Fasting}}\;{\text{glycemia}}\; ( {\text{mmol/l)}}\times{\text{Basal}}\;{\text{insulin}}\;(\upmu {\text{U/mL)}}}}{ 2 2. 5}$$

### Oral glucose tolerance test (oGTT)

For oGTT, the individuals were seen in the hospital in the morning after 10 h of fasting. Blood samples were collected prior to the administration of 75 g of anhydrous glucose dissolved in 300 mL of water orally (time 0′) and then every 30 min (times 30′, 60′, 90′ and 120′) for dosing of serum glucose and insulin.

### Quantification of serum cytokines

Blood samples were collected in BD Vacutainer^**®**^ SST™ II *Advance* tubes, centrifuged, the serum was separated and frozen in aliquots at − 70 °C for subsequent dosing. Serum Cytokines TNF-α, IL-6 e IL-1β they were quantified using enzyme immunoassay (ELISA) (BD Biosciences, New Jersey, EUA). Individuals in the FPLD and Control groups were fasted for 8 h before blood was withdrawn.

### Puncture of the subcutaneous fat

The subcutaneous adipose tissue of the neck was collected by puncture during aesthetic procedure. The procedure was performed by personnel trained under local anesthesia (2% lidocaine with epinephrine). After antisepsis with 2% chlorhexidine, puncture was performed with a 16G hypodermic needle (1.6 × 40 mm) attached to a 10 mL syringe. After the needle was introduced, repeated movements were made in tangential directions with the piston of the syringe drawn to cause vacuum. Approximately 1.0 mL of tissue (approximately 1 g) was collected which was frozen in liquid nitrogen and stored in a freezer at − 70 °C until processing. Five patients with FPLD and four healthy individuals matched for age and BMI were submitted to the procedure. In this study we had only 5 samples of adipose tissue from FPLD patients due to the non acceptance of 10 patients to perform the biopsy to collect the sample. Prior to the procedure, all subjects were asked to fast for 8 h.

### Human unfolded protein response PCR-array

RNA extraction was performed using the aspirated material from the cervical region. Briefly, the total adipose tissue that was collected was ground and homogenized following the extraction protocol of the RNeasy Lipid Tissue Mini Kit—QIAGEN. After quantification and evaluation of RNA integrity, a pool of samples from the CTRL group (n = 4) and the FPLD group (n = 5) were created. To do this, he added 100 ng of RNA from the CTRL and FPLD groups of each individual to make two sets of samples. We quantified the RNA concentration again and the following concentrations were observed: CTRL Pool 105.1 ng/μL and FPLD Pool 110.7 ng/μl. Next we produced cDNA using the iScript cDNA Synthesis Kit—Bio-Rad. The cDNA was then used in the Human Unfolded Protein Response PCR Array-Qiagen (PAHS-089Y). This first evaluation was performed as a screening, because it is a panel with 84 genes involved in unfolded protein response, which would later be validated in real-time PCR. The temperatures and times used to program the Bio-Rad unit (CFX96) were: 1 cycle—10 min—95 °C; 40 cycles—15 s—95 °C; Cycle 40—1 min—60 °C.

### Gene expression analysis

Briefly, 5 samples of adipose tissue from the FPLD group and 4 samples from the CTRL group were collected, and total RNA was extracted as described above. We used 1 μg of total RNA from each sample to produce cDNA using iScript cDNA Synthesis Kit (Bio-Rad). Quantitative PCR was performed using the SsoFastTM EvaGreen Supermix (Bio-Rad) and gene expression was quantified by real-time PCR in an CFX96 (Bio-Rad) (FPLD n = 5 and CTRL n = 4). Relative gene expression was normalized to GAPDH mRNA levels. The primers used in the qPCR analyzes are presented in Additional file 1: Table S1 (Primers Sequence). Specificity of amplification was tested by melting analysis. All samples were analyzed in duplicate.

### Statistical analysis

All results are expressed as mean ± SEM for metabolic experiments, gene expression and interleukin quantification experiments. t *Student* test was used to determine p values. Statistical significance was defined as p < 0.05.

## Results

### Familial partial lipodystrophy of the Dunnigan type induces metabolic changes

**Fig. 1:**
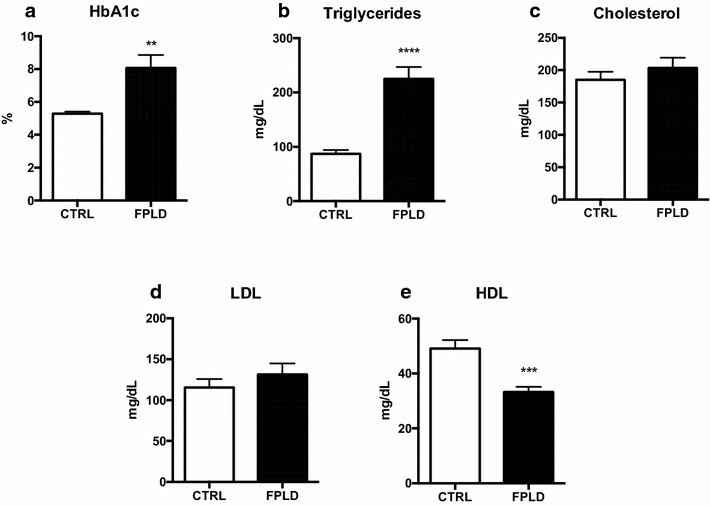
Lipodystrophy induces changes in serum levels of various blood metabolites. Individuals with FPLD have high blood metabolic parameters in comparison with control subjects. **a** Quantification of glycated hemoglobin levels. Student’s t test **p < 0.01. **b** Quantification of serum triglyceride levels. Student’s t test ***p < 0.005. **c** Evaluation of the amount of blood cholesterol. Student’s t test (ns). **d** Quantification of serum LDL levels. Student’s t test (ns). **e** Quantification of HDL levels. Student’s t test ****p < 0.001. Control group (n = 13), FPLD group (n = 14)

In our study, we observed that patients with FPLD had elevated glycosylated hemoglobin levels compared to normal subjects (8.1 ± 2.7%) (Fig. 1a), corroborating the results of plasma glucose levels, which also presented higher serum levels (130.9 ± 51.9 mg/dL) compared to control subjects (80.3 ± 7.3 mg/dL) (Table 1). We also observed higher levels of triglycerides (224.8 ± 85.9 mg/dL) (Fig. 1b), but no changes in LDL cholesterol levels (131 ± 51.6 mg/dL) (Fig. 1d). We observed a slight increase in total cholesterol (203.4 ± 61.0 mg/dL) (Fig. 1c), followed by reduction of HDL cholesterol (33.2 ± 7.3 mg/dL) (Fig. 1e). These data show metabolic alterations in people with FPLD, confirmed with a 64.2% prevalence of metabolic syndrome in this group. The comparison between the FPLD and the control groups showed that there were no significant differences in BMI, waist circumference and age among the members of the group, however, fasting serum insulin levels were higher in the FPLD group (42.4 ± 21.5 pmol/L) compared to the control group (26.3 ± 10.3 pmol/L) (Table 1).

**Table 1:** Clinical phenotype of FPLD subjects

	FPLD group	Control group	Normal reference	p value
	(mean/SD)	(mean/SD)	(mean)	FPLD vs. control
Age (years)	39.2 ± 14.29	31.3 ± 6.9	–	ns
BMI (kg/m^2^)	23.9 ± 2	23.5 ± 1.7	18.5–24.9	ns
Abdominal circumference (cm)	77.6 ± 5.2	75.3 ± 5.5	88	ns
Glycemia (mg/dL)	130.9 ± 51.9	80.3 ± 7.3	100	0.002
Insulin	42.4 ± 21.5	26.3 ± 10.3	25	0.02
HOMA-IR	1.1 ± 1.0	0.4 ± 0.2	1.7–2.0	0.03
HOMA-β	40.9 ± 35.5	131.5 ± 90.7	81.7	0.01

### Individuals with FPLD have a higher prevalence of IR

**Fig. 2:**
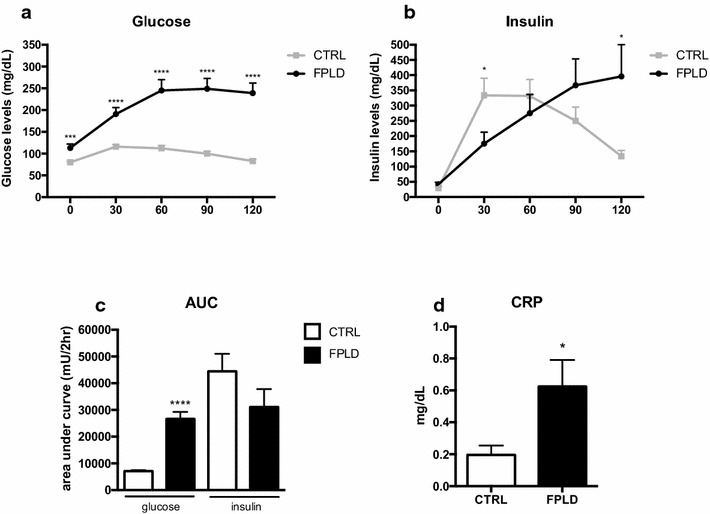
Oral Glucose Tolerance Test demonstrates the uncontrolled glycemic regulation and reduced insulin secretion. **a** Graph showing glucose levels during oGTT. The individuals in the FPLD group presented increased glucose levels in compared to control group. ***p < 0.005, ****p < 0.001. **b** Quantification of the insulin secretion during OTG. The FPLD group presented delayed insulin secretion compared to the control group *p < 0.05. **c** Area under the curve of oGTT graphs (plasma glucose and insulin). The AUC of glucose graph showed that individuals of FPLD group have higher AUC in compared to control group, demonstrating the uncontrolled glycemic; however no observed differences in insulin AUC. Student’s t test ****p < 0.001. **d** Evaluation of serum C-Reactive Protein levels. Student’s t test *p < 0.05. Control group (n = 13) and FPLD group (n = 14)

When assessing the values found in the HOMA-IR calculation, we observed an increase in the FPLD group compared to the control group, whereas HOMA beta values were significantly lower in the FPLD group compared to the normal group (Table 1). To confirm HOMA-IR results, we evaluated the blood glucose curves of oGTT from both groups. As a result, we obtained higher levels of glucose at all times in individuals with FPLD compared to the control group (Fig. 2a), better visualized in the quantification of the area under the glucose curve (AUCg) (Fig. 2c). We also measured the insulin levels of the different groups and found that individuals with lipodystrophy had higher insulin values at 30 and 120 min, but lower levels in the first period of oGTT (Fig. 2b), characterizing a state of hyperinsulinemia associated with loss of rapid and initial secretion of insulin. This pattern of insulin secretion resulted in an area under the insulin curve slightly lower than the control group. In addition, the FPLD group had low systemic subclinical inflammation, characterized by levels within the normal range of C-reactive protein (CRP) (0.62 ± 0.64 mg/dL), but higher than the control group (0, 20 ± 0.20 mg/dL) (Fig. 2d).

### Inflammatory profile of individuals with FPLD is altered compared to healthy subjects

**Fig. 3:**
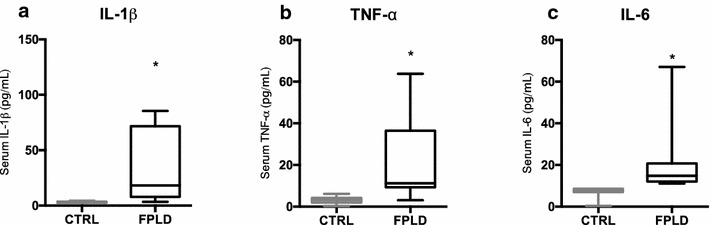
Interleukins responsible for the inflammatory profile are increased in the serum of individuals with FPLD. The levels of the main interleukins inflammatory process are altered in the serum of patients with FPLD. **a** IL-1β quantification in the serum of individuals with FPLD and control subjects. Student’s t test *p < 0.05. **b** Serum TNF-α quantification of FPLD individuals and control. Student’s t test *p < 0.05. **c** Measurement of IL-6 concentration in the serum of FPLD individuals and control. Student’s t test *p < 0.05. Control group (n = 13) and FPLD group (n = 14)

Faced with the evidence that individuals with FPLD had a subclinical inflammation, we evaluated the main interleukins responsible for systemic inflammation. The quantification of these interleukins in the serum of these individuals showed an increase in the proinflammatory cytokines, such as IL-1beta and TNF-alpha, which had high serum concentrations of FPLD individuals compared to control individuals (Fig. 3a, b). We also observed that IL-6 levels were elevated in the serum of FPLD subjects compared to healthy subjects (Fig. 3c).

### Subjects with FPLD present activation of ERS in adipose tissue

**Fig. 4:**
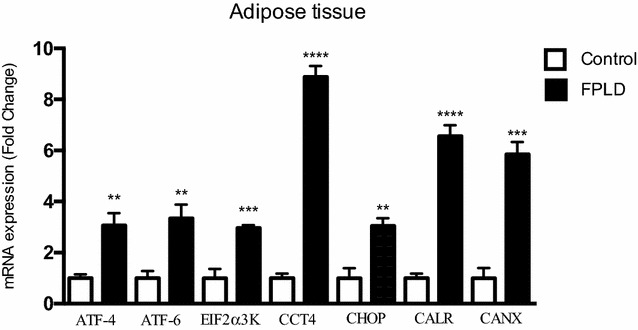
Metabolic changes in adipose tissue in FPLD subjects. Gene expression in adipose tissue in response FPLD. Control group (n = 4) and FPLD group (n = 5), paired with the same age and BMI. Total RNA was analyzed by real-time qPCR; data represented mean ± SEM. Student’s t test ****p < 0.001 versus control, **p < 0.01, ***p < 0.005

To evaluate genes involved in the ERS activation machinery in the adipose tissue of patients with FPLD and normal individuals (CTRL), initially, we used the array technique in which we evaluated the expression of 84 different genes involved in Unfolded Protein Response and ERS. This experiment was performed as a screening to guide the next steps, and showed that gene expression of CCT4, CALR and CANX chaperones were increased in patients with lipodystrophy compared to the CTRL group (Additional file 2: Fig. S1). These data were confirmed by real-time PCR and other genes involved in the activation of ERS were also evaluated in order to expand the evaluation. We observed that the expression of ATF-4, ATF-6, PERK and C/EBP Homologous Protein (CHOP), were also increased in patients with lipodystrophy compared to control subjects (Fig. 4).

## Discussion

In this study, we observed that patients with FPLD present disturbances in glycidic and lipid metabolism compared to healthy individuals, matched for age and BMI. We also observed insulin resistance in these individuals by both oGTT and HOMA-IR/β, and our data indicate an increase in plasma proinflammatory interleukins in patients with FPLD. In addition, we demonstrated the presence of ERS activation in the adipose tissue of individuals with FPLD compared to controls. Thus, we sought to demonstrate the relationship between metabolic abnormalities and the inflammatory profile with the expression of ERS-related genes.

The scarcity of clinical and molecular studies involving individuals with FPLD can be justified by the fact that this is a pathological condition considered rare and therefore, there is great difficulty in finding people with FPLD and obtaining biological samples to be analyzed. Thus, in our study, the limited number of adipose tissue samples used for the analysis of gene expression was caused by the small number of patients who accepted to undergo the aesthetic procedure to obtain adipose tissue. However, due to the rare number of people with FPLD, it is believed that the analyzed samples may provide very important information for understanding FPLD and how it induces dyslipidemia and insulin resistance. Another limitation of our study is that molecular evaluation was performed on subcutaneous adipose tissue of the neck and, therefore, may not represent other sites of subcutaneous tissue, usually more involved with insulin resistance, inflammation and activation of ERS, such as the abdomen.

Insulin resistance (IR) is the common pathophysiological component in obesity, metabolic syndrome and particularly type 2 diabetes mellitus, leading to varying degrees of altered glucose tolerance and may be manifested clinically by hyperglycemia and/or hyperinsulinemia. Insulin with its metabolic effects or growth promoters is the main anabolic hormone in the human body. Its action, which depends on the correct binding of the hormone to its receptor on the plasma membrane of the cells, involves a complex response that affects the metabolism of lipids, protein and carbohydrate [19]. In the other hand, the reduction of body fat especially in cases of lipodystrophy, the presence of insulin resistance becomes a primary factor for metabolic manifestations of this disease. The FPLD is a heterogeneous group of genetic disorders characterized by marked loss of subcutaneous fat of the extremities [1]. Patients with lipodystrophy revealed phenotypes that can be linked to autosomal dominant or recessive mutations. Among the autosomal dominant mutations, three loci of candidate genes were identified: the gene encoding the nuclear lamin A/C (*LMNA*), the gene of peroxisome proliferator activated receptor gamma *(PPARG*) and the gene encoding protein kinase B v-Akt—*murine thymoma oncogene homolog 2* (*AKT2*) [20]. In this study, it was observed that individuals with FPLD had higher insulin resistance, as evidenced by HOMA-IR calculation, as well as the calculation of AUCg, obtained by oGTT. The estimate of IR and function of the pancreatic β cells by the HOMA method is derived from a feedback model at baseline equilibrium condition [21]. The HOMA-β and AUCi values, obtained by patients with lipodystrophy, prove inadequate insulin secretion capacity. The state of IR in normal situations determines the compensatory increase in insulin secretion by the pancreas, which can be observed in obesity, however, not always detects the development of glycemic alterations [22]. To evolve with abnormal glucose levels, as observed in diabetic patients and specifically in this case in individuals with FPLD, there must be a decrease in insulin secretion by the pancreas, resulting in a relative insulin deficiency, manifested by loss glucose tolerance [19, 23].

Due to the simplicity of calculating the HOMA index, this has been increasingly used in clinical practice for the assessment of RI, requiring the determination of the cut-off points of the population, for example, the cut-off point defined by the BRAMS study [24], which evaluated Brazilian individuals of São Paulo State, detecting that the cutoff point for this population is 2.7 for the diagnosis of IR. The HOMA-IR of patients with lipodystrophy showed values below the cutoff point suggested by the Brazilian study, and since there are no cuts of HOMA-IR and HOMA-β for patients with this disease, we used the comparison with the control group, composed of individuals which showed no alterations in the distribution of body fat, observing that the group of patients with FPLD had higher IR and less secretion of capacity by pancreatic β cells. Corroborating this data, individuals with FPLD have metabolic disorders that induce an increase in triglyceride levels, fasting glycemia and low levels of HDL, which are diagnostic criteria for the metabolic syndrome.

The IR states are accompanied by lipotoxicity and secretion of adipokines [25, 26], which converge towards the development of metabolic inflammation [27]. The DM proinflammatory state is well known, being the adipose tissue adipokines potent inflammatory mediators [28] and sensitive to stimuli influenced by metabolic control [29–32]. In our results lipodystrophy patients did not show a systemic inflammatory state, when we evaluated the CRP levels. However, since we did not evaluate the ultrasensitive CRP, which is related to the development of cardiovascular diseases [33], we believe that the normal levels of CRP seen in patients with FPFD can be explained by the fact that we did not perform more sophisticated dosages which are usually used by other authors. Though, in order to deepen our studies on the inflammatory profile of lipodystrophy we analyzed the plasma levels of cytokines involved in the development of IR such as IL-1β, IL-6 e TNF-α [28, 34]. Our data show that individuals with FPLD develop chronic and subclinical systemic inflammation due to elevations in IL-1β, TNF-α and IL-6 concentrations. Thus, our data suggest that the increased inflammatory process induced by these cytokines could contribute to the exacerbation of insulin resistance in individuals with FPLD.

Hyperglycemia and increase of inflammatory cytokines present in DM, can induce activation of the ERS in various cell types, including the pancreatic beta cells, triggering their apoptosis [35]. The ER has multiple cellular functions mainly folding, assembly and formation of disulfide bonds of proteins. The Unfolded Protein Response (UPR) is a mechanism that is activated by the high concentration of proteins in the ER resulting immature protein degradation, decreasing the translation rate, and the increase of chaperones. Our data on gene expression of key proteins related to ERS in adipose tissue of individuals with FPLD demonstrated the hyperactivation of the UPR. These data suggest that the existence of molecular and metabolic disorders in adipocytes of individuals with FPLD is associated with the modulation of ERS, which may explain the insulin resistance and hyperglycemia in these patients.

The major membrane proteins of the ER involved in induction of UPR such as eIF2a3K (*RNA*-*activated protein Kinase*-*like ER Kinase*) and ATF-6 (*Activating Transcription Factor 6*) [36], showed increased gene expression in adipocytes of patients with FPLD characterizing the state of ERS. PERK activation in the UPR results in inhibition of translation of mRNAs, but paradoxically some mRNAs encoding proteins of adaptation to stress become relevant such as ATF-4 (*Activating Transcription Factor 4*) [37]. Furthermore activation ATF-4 by PERK pathway leads to increased expression of CHOP that retains the cell cycle and induce apoptosis [38].

The increase in expression of calreticulin and calnexin can be explained by the imbalance caused by the differences in intracellular calcium concentration that affects the secretory machinery of the ER [12]. In the case of increased expression of CCT4 (*Chaperonin Containing TCP1 subunit 4*) in adipose tissue of patients with FPLD it can be characterized by the necessity of raising the recovery rate of malformed proteins due to ERS, because these chaperones have the function of assisting the correct folding of proteins by ATP hydrolysis [39, 40]. These chaperones are also necessary for the folding of tubulin and actin in cells [41] besides being connected directly to many newly synthesized polypeptides in mammalian cells [42].

Thus, the activation of ERS in adipose tissue of patients with FPLD could be responsible for the organic response to metabolic stress inducing a severe metabolic syndrome seen in these patients compared to healthy control subjects. To our knowledge, this is the first study evaluating the ERS activation in FPLD patients, but since we studied a small sample, besides FPLD is a rare clinical situation, our findings shall be confirmed with further studies.

## Conclusions

In conclusion, this study demonstrated that Familial Partial Lipodystrophy of the Dunnigan type induces important metabolic disorders such as hyperglycemia, insulin resistance and hypertriglyceridemia. These damaging clinical environment are associated to increased systemic inflammatory profile and activation of the ERS in the subcutaneous adipose tissue, supporting the importance of fat deposits in the correct places of the human body. The discoveries of the findings may provide new routes to innovation of treatment providing a new perspective for the control of metabolic syndrome in this pathology.

## Additional files

**Additional file 1: Table S1.** Primers sequence.

**Additional file 2: Figure S1.** Human Unfolded Protein Response PCR-Array Layout in adipose tissue in individuals with FPLD. Regulation of genes expression in adipose tissue in response FPLD. (A) Heatmap representing the quantitation of 84 genes of ERS expressed in the subcutaneous adipose tissue of control group and FPLD group. (B) Quantitation of 84 genes (Fold-Change) in subcutaneous fat. ERS important genes are over-expressed in adipocytes of individuals with FPLD. Total RNA from adipose tissue were isolated from control group (n = 4) and FPLD group (n = 5) as described in the Experimental Procedures.

## Abbreviations

FPLD: familial partial lipodystrophy of the Dunnigan type
HbA1c: glycated hemoglobin
LDL-C: lower-density lipoprotein cholesterol
HDL-C: high-density lipoprotein-cholesterol
TG: triglyceride
AUC: area under curve
oGTT: oral glucose tolerance test
ERS: endoplasmic reticulum stress
ATF-4: activating transcription factor 4
ATF-6: activating transcription factor 6
EIF2α3K: eukaryotic translation initiation factor 2-alpha kinase 3
CCT4: chaperonin containing TCP1 subunit 4
CHOP: C/EBP Homologous Protein
CALR: calreticulin
CANX: calnexin
IL-1β: interleukin 1 beta
TNF-α: tumor necrosis factor alpha
IL-6: interleucina 6
DM: diabetes mellitus
HOMA-IR: homeostasis model of assessment insulin resistance
HOMA- β: homeostasis model of assessment β cells function
