# Supplementary figures and images for: Endoplasmic reticulum stress activation in adipose tissue induces metabolic syndrome in individuals with familial partial lipodystrophy of the Dunnigan type
Source: Diabetol Metab Syndr. 2018 Feb 9;10:6. doi: 10.1186/s13098-017-0301-6 (PMC5807843; doi:10.1186/s13098-017-0301-6)

**B**


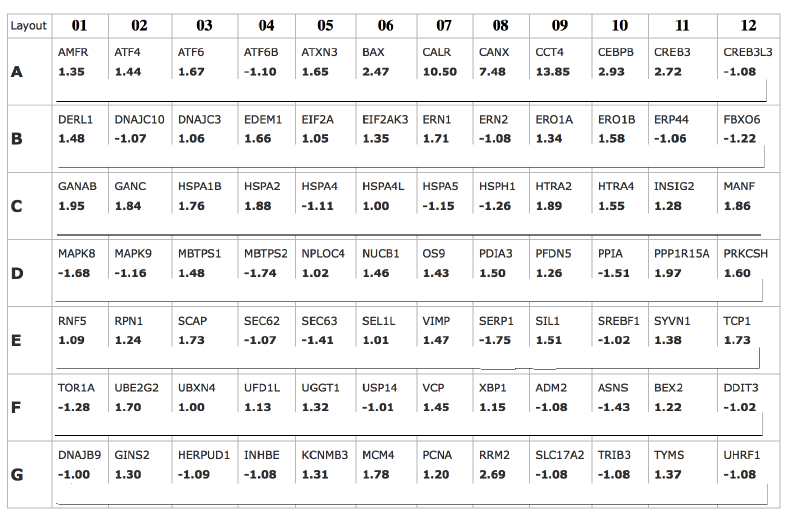

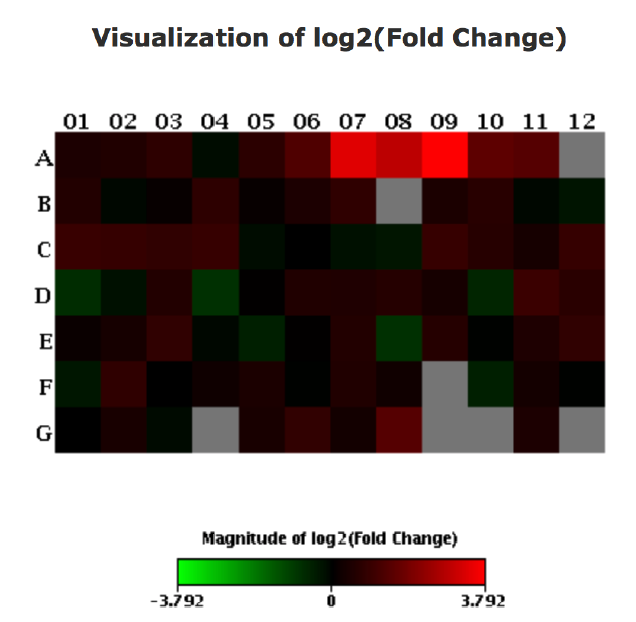


**A**

**Figure. S1.**

Supplement: Supplementary file 2 — Additional file 2: Figure S1. Human Unfolded Protein Response PCR-Array Layout in adipose tissue in individuals with FPLD. Regulation of genes expression in adipose tissue in response FPLD. (A) Heatmap representing the quantitation of 84 genes of ERS expressed in the subcutaneous adipose tissue of control group and FPLD group. (B) Quantitation of 84 genes (Fold-Change) in subcutaneous fat. ERS important genes are over-expressed in adipocytes of individuals with FPLD. Total RNA from adipose tissue were isolated from control group (n = 4) and FPLD group (n = 5) as described in the Experimental Procedures. [file 13098_2017_301_MOESM2_ESM.docx]
